# Fatty acid composition of birds and game hunted by the Eastern James Bay Cree people of Québec

**DOI:** 10.3402/ijch.v75.30583

**Published:** 2016-08-04

**Authors:** Francoise Proust, Louise Johnson-Down, Line Berthiaume, Karine Greffard, Pierre Julien, Elizabeth Robinson, Michel Lucas, Éric Dewailly

**Affiliations:** 1 Axe Santé publique et pratiques optimales en santé, Centre de recherche du Centre Hospitalier Universitaire (CHU) de Québec, Québec, QC, Canada; 2 Department of Social and Preventive Medicine, Université Laval, Québec, QC, Canada; 3 School of Dietetics and Human Nutrition, McGill University, Montréal, Québec, QC, Canada; 4 Lipid Laboratory, Centre de recherche du Centre Hospitalier Universitaire (CHU) de Québec, Québec, QC, Canada; 5 Public Health Department of the James Bay Cree Territory, Cree Board of Health and Social Services of James Bay, Québec, QC, Canada

**Keywords:** wild game, wild birds, food composition, food analysis, partridge, goose, beaver, moose, caribou, bear

## Abstract

**Background:**

Indigenous peoples have traditionally relied on foods hunted and gathered from their immediate environment. The Eastern James Bay Cree people consume wild game and birds, and these are believed to provide health as well as cultural benefits.

**Objective:**

To determine the fatty acid (FA) composition of traditional game and bird meats hunted in the Eastern James Bay area.

**Design:**

Harvested traditional game and birds were analysed for FA composition. A total of 52 samples from six wildlife species were collected in the areas of Chisasibi, Waswanipi and Mistissini, of which 35 were from birds (white partridge and Canada goose) and 17 were from land animals (beaver, moose, caribou and black bear).

**Results:**

Alpha-linolenic acid (ALA) was the most common n-3 polyunsaturated fatty acid (PUFA) in all samples except for the black bear flesh, in which it was docosapentaenoic acid (DPAn-3). In white partridge, beaver and caribou flesh, PUFAs (mainly n-6) were the most common category of fats while in goose, moose and black bear flesh, monounsaturated fatty acids (MUFAs) predominated. In all species, saturated fatty acids (SFAs) were the second most important FAs. It would appear that in the land animals and birds that were analysed, the SFA content was lower and the PUFA content was higher than store-bought meats giving them a more heart-healthy profile.

**Conclusions:**

These results showed that the FA composition of game species consumed by the James Bay Cree population is consistent with a beneficial diet and that traditional foods should continue to be promoted among the Cree people to provide better physical health as well as social and spiritual benefits.

Traditional food obtained through hunting and fishing not only provides necessary nutrients but also contributes to the social, cultural and spiritual values associated with the consumption of food items originating from the environment. They are minimally processed (1) and are associated with a better dietary pattern, that is, fewer poor nutritional quality market foods (2–4). They are, therefore, a vital component of the health and well-being of Aboriginal Canadian populations (5). The traditional Cree diet is composed of marine or freshwater fish species, large and small game (land mammals and birds) and wild berries (6,7)

Contacts with Europeans led to the introduction of imported foods such as sugar, white flour and lard which were originally beneficial by staving off famine but more recently have contributed to the emergence of previously unknown diseases in this population, such as type 2 diabetes (7). Since the mid-20th century, the diet and eating habits of the Cree people have changed dramatically due to greater accessibility of purchased or market foods. The availability of market foods has led to a lower contribution of traditional foods to the total dietary energy intake among Eastern James Bay Cree peoples (8).

The nutrient composition of fish species consumed by Northern indigenous peoples is relatively well documented (9,10); these species are known to be naturally rich in omega-3 polyunsaturated fatty acids (n-3 PUFAs) that are protective against cardiovascular disease (CVD) (11,12). However, despite their importance in the traditional Cree diet, only sparse data on fatty acid (FA) composition are available for terrestrial and avian game species. Therefore, documenting FA composition of frequently consumed game species is important to evaluate the dietary FA sources and consequently their contribution to the blood FA profile measured in the inhabitants of Eastern James Bay Cree region.

Thus, in this study, we analysed wild bird and land mammal species frequently harvested and consumed by the Cree residents of the Eastern James Bay Territory in order to characterize their FA composition.

## Methods and materials

The project was undertaken as part of the *Multi-community Environment and Health Study in Iiyiyiu Aschii* survey carried out in the Eastern James Bay Cree population, a joint project of the Cree Board of Health and Social Services of James Bay (CBHSSJB), Laval, McGill and McMaster Universities (13–15). Previous research agreements were in place with the Cree Nations of Chisasibi, Waswanipi and Mistissini, and local authorities were informed of the data collection.

### Study area

**Fig. 1.**
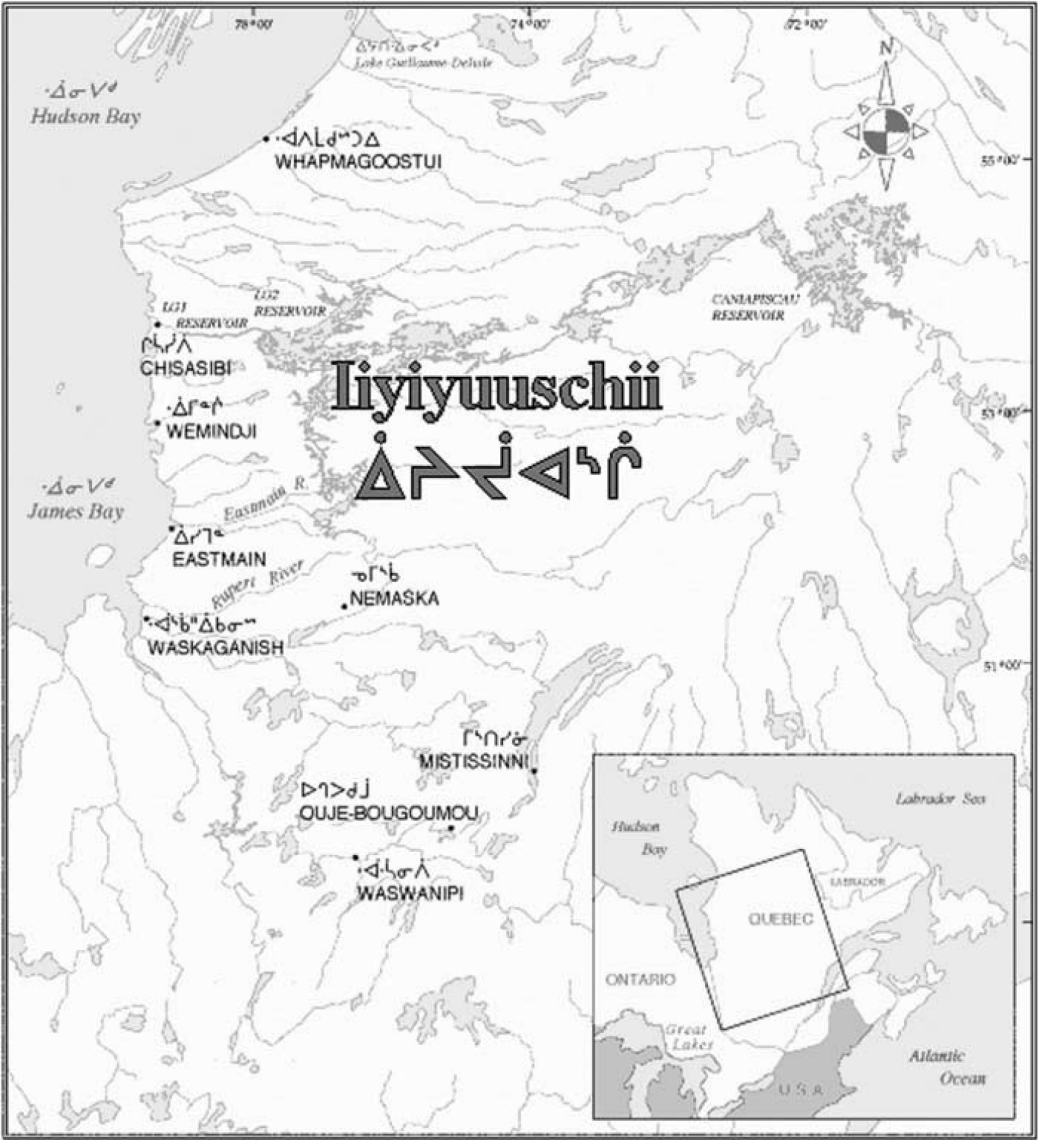
Map of the James Bay Cree communities, Québec (Canada).

Field research to collect samples of wildlife species was conducted in 2011, 2012 and 2014 in the areas of Chisasibi, Waswanipi and Mistissini, three of the nine Eastern James Bay Cree communities. Waswanipi and Mistissini are inland communities located in the south-eastern James Bay Territory, whereas Chisasibi is located further north on the eastern coast of the James Bay (Fig. 1). The Eastern James Bay territory, called *Eeyou Istchee*, is located between the 49th and the 55th parallel in the province of Québec (Canada) and covers more than 350,000 km^2^. In 2011, the total indigenous population of the Eastern James Bay territory was 16,010 (16).

### Sample collection

A total of 52 samples from six wildlife species were collected, of which 35 were birds (white partridge and Canada goose) and 17 were land animals (beaver, moose, caribou and black bear). The parts of the animals that were sampled were those usually consumed by the *Eeyouch* (Cree people).

Local research assistants were trained in the safe handling of harvested meats using information from elders, the Ministry of Agriculture and Fisheries and Food of the province of Québec (17). Sampling took place in the homes of hunters and participation was voluntary. Samples were collected from fresh or frozen kills, and the animals and the precise location of those samples on the carcasses were randomly selected. Once collected, samples were identified and stored at −20°C for delivery to the Lipid Laboratory of the “Centre Hospitalier de l'Université Laval” for analysis. The skin and flesh of the partridge and geese were separated to allow a separate assessment of their FA content. Flesh, fat, skin and organ meats such as liver, heart, kidney, stomach and eggs were analysed.

### FA analysis

Samples were analysed individually for FA composition. Most samples were raw except for two samples of caribou muscle and fat, which were smoke dried, and three samples of rendered bear fat. Rendered bear fat is subcutaneous fat that is melted down after which all flesh is removed and frozen for storage.

Approximately 50 mg of the samples were homogenized using a glass potter. Lipids were extracted along with a C(15) phosphatidylcholine as an internal standard (Avanti Polar Lipids, 211 Alabaster, AL), in a chloroform/methanol (C–M) mixture (2:1 vol/vol) according to a modified Folch method (18). Total lipids were then methylated using methanol/benzene (4:1 vol/vol) and acetyl chloride (19). The FA profiles were obtained by capillary gas chromatography using a temperature gradient on an HP 5890 gas chromatograph (Hewlett Packard, Toronto, Canada) equipped with an HP-88 capillary column (100 m×0.25 mm ID×0.20 µm film thickness; Agilent Technologies, Santa Clara, CA) coupled with a flame ionization detector (FID). Helium was used as the carrier gas (split ratio of 1:40). FAs were identified according to their retention time, using a standard mixture of FA as a basis for comparison (FAME 37 mix, Supelco Inc., Bellefonte, PA) – the C15:0 as well as the following FA – C22:5n-6 (Larodan AB, Malmö, Sweden), C22:5n-3 cis-12 (Supelco Inc.) and a mixture of 31 FA GLC-411 (NuChek Prep Inc., Elysian, MN). Finally, a mixture of trans FA containing C18:2n-6 cis/trans (Supelco Inc.), C18:3n-3 cis/trans (Supelco Inc.) and C14:1 trans-9, C16:1 trans-9 and isoforms of C18:1 (cis-6, cis-11, cis-12, cis-13 trans-6 and trans-11) (Supelco Inc.) were also used as standards. Results were expressed as percent of total FAs and as grams of FAs per 100 g of edible portion. A precision of 0.01 g/100 g and 0.1% was obtained when less than three samples were collected.

### Statistical analysis

Means and standard deviations were calculated using Microsoft Excel and SAS 9.2 (2008, SAS Institute Inc., Cary, NC).

## Results and discussion

White partridge, beaver and caribou flesh had higher quantities of PUFAs (mainly n-6), whereas goose, moose and black bear flesh had monounsaturated fatty acids (MUFAs). PUFA proportions ranged from 44 to 57% of total FAs when PUFAs predominated and MUFAs were in the range of 41 to 55% when MUFAs predominated, while saturated fatty acids (SFAs) accounted for approximately 30–40% of total FAs in all the species analysed (Tables I and II). In all species, SFAs were the second most common FAs.

**Table:** *Table I*. Mean concentrations (±SD) of FAs in samples of birds traditionally consumed by the Eastern James Bay Cree people expressed in % of total FAs and g/100 g edible portion

	White partridge						Canada goose					
		
	Flesh, raw	Fat, raw	Skin, raw	Liver, raw	Heart, raw	Stomach, raw	Flesh, raw	Fat, raw	Skin, raw	Liver, raw	Heart, raw	Eggs, raw
n	4	1	1	1	1	1	6	5	5	1	5	3
Total FAs												
g/100 g	1.34±0.575	55.0	9.89	4.42	1.80	1.11	4.71±0.845	69.5±8.56	65.1±9.46	4.57	4.04±0.979	4.81±1.48
Total SFAs												
%	43.2±17.9	20.0	42.5	42.3	36.3	40.5	32.9±2.20	34.5±1.57	32.4±2.22	38.1	33.0±0.932	34.9±0.212
g/100 g	0.510±0.0914	11.0	4.20	1.87	0.653	0.451	1.55±0.273	24.0±3.83	21.0±2.71	1.74	1.33±0.308	1.68±0.505
Palmitic acid (16:0)												
%	29.3±18.4	11.7	26.0	13.5	15.0	15.9	21.0±1.58	24.4±0.806	23.1±1.37	21.2	20.7±0.961	22.7±0.726
g/100 g	0.220±0.0491	6.41	2.57	0.596	0.270	0.178	0.990±0.194	17.0±2.48	15.0±1.93	0.969	0.843±0.236	1.10±0.354
Stearic acid (18:0)												
%	22.6±10.1	7.00	11.9	27.2	19.6	22.6	11.2±1.74	9.41±0.864	8.64±1.44	16.0	11.5±1.42	11.2±0.696
g/100 g	0.264±0.0561	3.85	1.17	1.20	0.353	0.252	0.526±0.0939	6.59±1.29	5.60±1.06	0.734	0.453±0.0641	0.532±0.150
Total MUFAs												
%	9.35±1.99	7.73	16.0	2.18	10.4	5.39	43.8±4.30	51.1±6.05	52.9±4.41	26.6	37.7±5.51	45.3±1.41
g/100 g	0.113±0.0683	4.25	1.58	0.0966	0.186	0.0601	2.04±0.272	35.8±7.47	34.7±7.10	1.21	1.53±0.445	2.17±0.647
Oleic acid (18:1n-9)												
%	6.01±1.89	5.59	13.2	1.23	6.67	2.44	37.2±3.98	44.7±5.91	46.3±4.61	22.2	31.3±4.57	39.3±1.36
g/100 g	0.0865±0.0507	3.07	1.31	tr	0.120	tr	1.73±2.31	31.4±6.86	30.4±6.35	1.01	1.27±0.354	1.89±0.575
Total PUFAs												
%	47.5±16.3	72.3	41.6	55.5	53.3	54.1	23.6±5.58	14.9±7.14	15.1±5.71	35.4	29.7±5.85	20.3±1.58
g/100 g	0.703±0.426	39.7	4.11	2.46	0.959	0.603	1.13±0.460	9.96±3.33	9.68±3.04	1.62	1.20±0.400	0.984±0.323
Total n-3 FAs												
%	4.69**±**2.77	15.5	5.54	4.74	5.67	5.82	5.13**±**4.38	5.13**±**5.71	4.50**±**4.51	14.0	5.99**±**5.08	5.47**±**3.05
g/100 g	0.0730**±**0.0505	8.52	0.548	0.210	0.102	0.0649	0.268±0.285	3.25±3.15	2.82±2.55	0.639	0.257±0.272	0.293±0.277
ALA (18:3n-3)												
%	3.00**±**2.22	15.2	5.54	2.26	3.84	1.41	4.25**±**4.27	5.13**±**5.72	4.50**±**4.51	9.96	4.64**±**4.70	3.51**±**2.72
g/100 g	tr	8.33	0.548	0.100	0.0692	tr	0.227±0.272	3.25±3.15	2.82±2.55	0.455	0.203±0.250	0.195±0.187
EPA (20:5n-3)												
%	0.396±0.0811	0	0	0.731	0.462	1.27	0.193±0.145	0	0	1.61	0.385±0.312	0.319±0.176
g/100 g	tr	0	0	tr	tr	tr	tr	0	0	0.0734	tr	tr
DPAn-3 (22:5n-333)												
%	0.668±0.404	0.197	0	0.649	0.583	0.929	0.239±0.116	0	0	0.970	0.396±0.136	0.650±0.280
g/100 g	tr	0.108	0	tr	tr	tr	tr	0	0	tr	tr	tr
DHA (22:6n-3)												
%	0.620±0.511	0	0	0.929	0.631	2.02	0.388±0.102	0	0	1.34	0.546±0.155	0.872±0.130
g/100 g	tr	0	0	tr	tr	tr	tr	0	0	0.0614	tr	tr
Total n-6 FAs												
%	42.8**±**13.7	56.8	36	50.8	47.7	48.3	18.5**±**2.68	9.76±1.62	10.6±1.38	21.4	23.7±2.60	14.9±2.87
g/100 g	0.630±0.377	31.2	3.56	2.25	0.857	0.538	0.865±0.181	6.70±0.655	6.86±7.72	0.977	0.943±0.158	0.691±0.130
LA (18:2n-6)												
%	31.2±11.1	55.8	34.2	34.70	35.90	28.90	13.5±1.85	9.50±1.58	10.4±1.26	8.91	13.4±1.58	7.14±0.34
g/100 g	0.466±0.303	30.7	3.39	1.53	0.646	0.322	0.640±0.167	6.51±0.596	6.71±0.741	0.407	0.542±0.152	0.340±0.0911
AA (20:4n-6)												
%	9.58±4.48	0.289	0.486	12.4	10.1	17.1	4.22±1.33	0	0.0270±0.0646	11.1	9.62±2.32	6.21±1.80
g/100 g	0.140±0.0838	0.159	tr	0.551	0.181	0.190	0.193±0.0433	0	tr	0.509	0.371±0.0198	0.284±0.0593

AA: arachidonic acid (20:4n-6); ALA: α-linolenic acid (18:3n-3); DHA: docosahexaenoic acid (22:6n-3); DPAn-3: docosapentaenoic acid (22:5n-3); EPA: eicosapentaenoic acid (20:5n-3)

FA: fatty acid; LA: linoleic acid (18:2n-6); MUFA: monounsaturated FA; n: number of samples; SFA: saturated fatty acid.

Total FAs represent the total of all FAs identified in the analyses.

Total SFAs=sum (14:0+16:0+18:0+20:0+22:0+24:0).

Total MUFAs=sum (14:1n5c;t+16:1n7c;t+18:1n5c+18:1n6c+18:1n7c;t+18:1n9c;t+18:1n11c;t+18:1n12c;t+20:1n9c;t+20:1n12c+22:1n9c+24:1n9c).

Total PUFAs=sum (18:3n-3+18:4n-3+20:3n-3+20:4n-3+20:5n-3+22:3n-3+22:5n-3+22:6n-3)+(18:2n-6+18:3n-6+20:2n-6+20:3n-6+20:4n-6+22:2n-6+22:4n-6+22:5n-6)+(18:3 n3t/t/t)+(18:2 n6t/t;c/t;t/c)).

Total n-3=sum (18:3n-3+18:4n-3+20:3n-3+20:n-64n-3+20:5n-3+22:3n-3+22:5n-3+22:6n-3+(18:3n3t/t/t)).

Total n-6=sum (18:2+18:3+20:2+20:3+20:4+22:2+22:4+22:5+(18:2n6t/t;c/t;t/c)).

FAs are cis unless noted otherwise.

**Table:** *Table II*. Mean concentrations (± SD) of FAs in samples of land mammals traditionally consumed by the James Bay Cree people expressed in % of total FAs and g/100 g edible portion

	Beaver		Moose	Caribou			Black bear			
			
	Flesh, raw	Fat, raw	Flesh, raw	Flesh, smoked dried	Fat, smoked dried	Heart, raw	Kidney, raw	Flesh, raw	Fat, raw	Fat, rendered
n	2	2	1	2	2	2	1	1	1	3
Total FAs										
g/100 g	1.05	37.3	1.14	1.17	57.2	26.2	1.70	1.40	75.5	73.1±5.22
Total SFAs										
%	29.8	21.2	36.2	31.0	48.9	53.9	40.7	32.7	34.7	34.5±0.922
g/100 g	0.303	7.73	0.413	0.299	28.0	16.4	0.694	0.459	26.2	25.2±1.72
Palmitic acid (16:0)										
%	15.5	14.1	19.1	12.4	17.4	26.6	17.3	24	26.4	26.9±1.69
g/100 g	0.157	5.12	0.217	0.146	9.94	8.13	0.295	0.336	19.9	19.6±0.964
Stearic acid (18:0)										
%	12.9	5.89	15.6	17.2	29.8	25.1	18.1	7.57	6.84	5.91±1.14
g/100 g	0.131	2.18	0.178	0.202	17.1	7.67	0.308	0.106	5.17	4.37±1.14
Total MUFAs										
%	13.8	21.8	41.3	25.5	46.2	31.2	18.6	54.5	64.6	62.5±1.24
g/100 g	0.135	8.02	0.471	0.299	26.4	9.13	0.317	0.764	48.8	45.7±4.45
Oleic acid (18:1n-9)										
%	8.52	13.5	33.9	20.5	41.5	26.8	13.9	41.7	53.8	52.4±1.92
g/100 g	0.0805	4.87	0.387	0.240	23.7	8.26	0.236	0.585	40.6	38.3±4.01
Total PUFAs										
%	57.1	58.2	23.3	43.9	5.23	15	40.7	12.8	0.658	2.67±1.53
g/100 g	0.620	22.1	0.265	0.516	2.99	0.703	0.693	0.179	0.497	1.91±0.980
Total n-3 FAs										
%	7.62	19.4	3.82	10.4	2.16	1.51	4.6	3.34	0.242	1.10**±**0.59
g/100 g	0.0770	7.11	0.0436	0.122	1.24	0.0842	0.0784	tr	0.183	0.79±0.38
ALA (18:3n-3)										
%	5.47	18.6	2.43	4.62	1.95	0.278	0.265	0.710	0.242	0.888**±**0.522
g/100 g	tr	6.82	tr	tr	1.12	tr	tr	tr	0.183	0.634±0.338
EPA (20:5n-3)										
%	0.245	0	0.557	2.53	0	0.236	0.988	0.163	0	0
g/100 g	tr	0	tr	tr	0	tr	tr	tr	0	0
DPAn-3 (22:5n-3)										
%	1.55	0.149	0.629	2.28	0	0.902	2.75	1.76	0	0.163±0.0349
g/100 g	tr	tr	tr	tr	0	tr	tr	tr	0	0.118±0.0218
DHA (22:6n-3)										
%	0.111	0	0.214	0.506	0	0.0624	0.483	0.705	0	0
g/100 g	tr	0	tr	tr	0	tr	tr	tr	0	0
Total n-6 FAs										
%	49.5	38.8	19.5	33.6	3.07	13.5	36.1	9.43	0.416	1.57±0.74
g/100 g	0.543	15.0	0.222	0.394	1.75	0.619	0.614	0.132	0.314	1.13±0.475
LA (18:2n-6)										
%	41.3	37.3	14.4	24.6	2.08	7.34	10.2	4.02	0.416	1.53±0.676
g/100 g	0.457	14.5	0.164	0.289	1.19	0.406	0.174	tr	0.314	1.10±0.433
AA (20:4n-6)										
%	5.60	0.182	4.01	7.14	0	5.2	23.2	4.12	0	0.0206±0.0409
g/100 g	tr	0.0693	tr	0.0840	0	0.142	0.395	tr	0	tr

AA: arachidonic acid (20:4n-6); ALA: α-linolenic acid (18:3n-3); DHA: docosahexaenoic acid (22:6n-3); DPAn-3: docosapentaenoic acid (22:5n-3); EPA: eicosapentaenoic acid (20:5n-3); FA: fatty acid; LA: linoleic acid (18:2n-6); MUFA: monounsaturated FA; n: number of samples; SFA: saturated fatty acid.

Total FAs represent the total of all FAs identified in the analyses.

Total SFAs=sum (14:0+16:0+18:0+20:0+22:0+24:0).

Total MUFAs=sum (14:1n5c;t+16:1n7c;t+18:1n5c+18:1n6c+18:1n7c;t+18:1n9c;t+18:1n11c;t+18:1n12c;t+20:1n9c;t+20:1n12c+22:1n9c+24:1n9c).

Total PUFAs=sum (18:3n-3+18:4n-3+20:3n-3+20:4n-3+20:5n-3+22:3n-3+22:5n-3+22:6n-3)+(18:2n-6+18:3n-6+20:2n-6+20:3n-6+20:4n-6+22:2n-6+22:4n-6+22:5n-6)+(18:3n3t/t/t)+(18:2 n6t/t;c/t;t/c)).

Total n-3=sum (18:3n-3+18:4n-3+20:3n-3+20:n-64n-3+20:5n-3+22:3n-3+22:5n-3+22:6n-3+(18:3n3t/t/t)).

Total n-6=sum (18:2+18:3+20:2+20:3+20:4+22:2+22:4+22:5+(18:2n6t/t;c/t;t/c)).

FAs are cis unless noted otherwise.

Palmitic acid (16:0) was the most common SFA except in caribou where the longer chain stearic acid (18:0) prevailed. Oleic acid (18:1) was the most common MUFA. ALA (18:3n-3) was the most common n-3 PUFA in all samples except for the black bear flesh where DPAn-3 was the most common FA. Overall, total n-3 PUFA levels were low except in the fat samples of the land mammals and the fat and skin samples of the wild birds. Linoleic acid (LA) (18:2n-6) was the most common n-6 PUFA, and the n-6 PUFAs were the predominant PUFA in all samples.

The results for goose are similar to the FA data previously reported for oven-roasted Canada goose by Belinsky and Kuhnlein (20). They found 2.52 g MUFA/100 g, 1.72 g SFA/100 g and 1.52 g PUFA/100 g in Canada goose flesh, and their higher values may be attributable to comparing cooked and raw samples (21). Canada goose is a commonly eaten food in the Eastern James Bay Cree region. It is harvested during the fall and spring goose hunts, two important cultural events that are very important traditional activities along the Eastern James Bay coast (22). It is one of the most commonly eaten traditional foods in this population (23).

There is great variation in the FA composition of game meats as sampled in Europe and North America (24,25). Our samples also showed disparity although the small number of samples did not allow us to do a statistical comparison. With the exception of wild boar, the total n-3 PUFA (3.34–10.4%) was inferior to that analysed by Valencak et al. in Europe (4.1–24.1%) (24). In wild game, total PUFA (0.179–0.620 g/100 g) was similar to that of Cordain et al. in North American species in Colorado (0.625–0.724 g/100 g) (25).

Because traditional game and birds are usually replaced by store-bought meats, we compared the total SFAs in the wild birds to chicken and the land mammals to beef as characterized on the Canadian Nutrient File (26). Total SFAs were lower in land mammal flesh than beef (0.30–0.46 g/100 g vs. 4.33 g/100 g) (26). In the pure fat, this was not so clear: there was 49.8 g of SFAs in beef compared with a range of 37.3 g in beaver to 75.5 g in bear. Total MUFA was lower in the wild mammals versus beef, and total n-3 PUFA was higher. In wild birds versus chicken, total SFAs (0.51–1.55 g vs. 2.20 g/100 g), MUFAs (0.11–2.04 g vs. 2.73 g/100 g) and PUFAs (0.70–1.13 g vs. 2.18 g/100 g) were lower in partridge and goose than chicken. For the skin, partridge had less SFAs, MUFAs and PUFAs than chicken, whereas goose had more. Similar to store-bought foods such as beef and chicken (26), trans FAs were present in small amounts. ALA and total n-3 were much superior in the wild birds. Overall, we can conclude that wild birds and game appear to be better sources of n-3 FAs than store-bought meats.

n-3 PUFAs have many physiological properties that would appear to decrease the risk of CVD and have been shown to do so in observational studies (12,27). Consumption of ALA has been associated with the reduction of risk and death from CVD in older adults and men in prospective cohorts (27,28). In addition, a meta-analysis showed consistent reduction of CVD with dietary ALA (29). In a pooled analysis of cohort studies, Jakobsen et al. found that replacing SFAs with PUFAs was beneficial for cardiovascular health, but MUFAs show no effect (30). Therefore, since SFAs were lower and PUFAs higher in the wild game and birds, replacing store-bought meats with wild game and birds could improve CVD health. Also, recent evidence confirmed the benefits of n-6 PUFAs as well as n-3 PUFAs (31–33).

We acknowledge that a more stringent sampling process and a greater number of samples would yield more representative results. The mixture of cooked and raw samples was another limitation of our study. However, other authors have found very low intraspecies variation (<5%) of PUFA content with gentle cooking that indicated limited intramuscular fat in game species (34). To our knowledge, this is the first time that samples of game meat from this region have been analysed for FA content, and the limitations of our study do not prevent the use of these rare species FA values.

## Conclusion

These results add information on FA profiles of foods that have not been previously studied. We have shown that the FA composition of game species harvested and consumed by the Eastern James Bay Cree people may contribute to a healthy diet due to their high proportions of n-3 PUFAs. Traditional wildlife foods such as the game species samples analysed in this study appear to be better choices as compared to lower quality store-bought foods.

Traditional foods also have cultural importance in addition to being good sources of high-quality protein and essential nutrients. For example, the Canada goose harvesting season, a cultural tradition that provides the Cree people with the opportunity for passing essential traditional knowledge to the next generation (20) helps ensure the continuation of social and cultural identity of the Eastern James Bay Cree.

A previous study carried out in seven James Bay Cree communities showed that older adults consumed traditional food more frequently than younger age groups (13–15,35). Concurrent with decreased traditional food intake in this indigenous population, diabetes and CVD have increased in alarming proportions (16). Hence, the consumption of traditional foods especially wild game and birds should continue to be promoted among the Cree population, particularly among younger individuals who are less likely to consume traditional foods.

Finally, this study gives the Cree communities a better understanding of some of the nutritional benefits of their traditional food species. This could aid health practitioners in the development of nutritional health promotion materials. In the future, further analysis of contaminants and persistent lipophilic pollutants and heavy metals such as mercury in these species is needed to better understand and mitigate the risks posed by possible environmental threats although this should always be balanced against the harm of replacing these nutrient-rich foods by store-bought foods.
